# Expert Algorithm for Substance Identification Using Mass Spectrometry: Application to the Identification of Cocaine on Different Instruments Using Binary Classification Models

**DOI:** 10.1021/jasms.3c00090

**Published:** 2023-05-31

**Authors:** Samantha A. Mehnert, J. Tyler Davidson, Alexandra Adeoye, Brandon D. Lowe, Emily A. Ruiz, Jacob R. King, Glen P. Jackson

**Affiliations:** † Department of Forensic and Investigative Science, West Virginia University, Morgantown, West Virginia 26506, United States; ‡ C. Eugene Bennett Department of Chemistry, West Virginia University, Morgantown, West Virginia 26506, United States

**Keywords:** spectral comparisons, spectral algorithm, search algorithm, forensic science, binary classification, drug identification

## Abstract

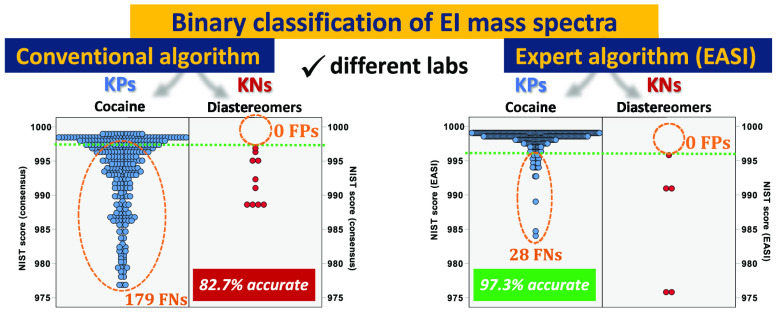

This is the second of two manuscripts describing how general linear modeling (GLM) of a selection of the most abundant normalized fragment ion abundances of replicate mass spectra from one laboratory can be used in conjunction with binary classifiers to enable specific and selective identifications with reportable error rates of spectra from other laboratories. Here, the proof-of-concept uses a training set of 128 replicate cocaine spectra from one crime laboratory as the basis of GLM modeling. GLM models for the 20 most abundant fragments of cocaine were then applied to 175 additional test/validation cocaine spectra collected in more than a dozen crime laboratories and 716 known negative spectra, which included 10 spectra of three diastereomers of cocaine. Spectral similarity and dissimilarity between the measured and predicted abundances were assessed using a variety of conventional measures, including the mean absolute residual and NIST’s spectral similarity score. For each spectral measure, GLM predictions were compared to the traditional exemplar approach, which used the average of the cocaine training set as the consensus spectrum for comparisons. In unsupervised models, EASI provided better than a 95% true positive rate for cocaine with a 0% false positive rate. A supervised binary logistic regression model provided 100% accuracy and no errors using EASI-predicted abundances of only four peaks at *m*/*z* 152, 198, 272, and 303. Regardless of the measure of spectral similarity, error rates for identifications using EASI were superior to the traditional exemplar/consensus approach. As a supervised binary classifier, EASI was more reliable than using Mahalanobis distances.

## Introduction

Gas chromatography–electron ionization–mass spectrometry (GC–EI–MS) is classified by the Scientific Working Group for the Analysis of Seized Drugs (SWGDRUG) and ASTM as both a Category A technique for mass spectrometry and a Category B technique for chromatography, based on its maximum potential discriminating power. As a hyphenated technique, GC–MS is therefore considered a confirmatory method because it uses structural information and is among the most selective and specific of analytical techniques.^1^ One of the earliest uses of GC–EI–MS to identify drugs of abuse in a forensic setting was in 1971 when Law et al. highlighted the ability of mass spectrometry to offer reliable mass spectral fingerprints for immediate and accurate identification of compounds.^2^ The specificity and selectivity of GC–EI–MS derive from the two-dimensional nature of the data; one can combine the mass spectral and retention time data to discriminate among structurally similar analogs.^3−14^ However, if a questioned sample has not, or cannot, be analyzed on the same instrument with the same conditions as a reference sample, the increased uncertainty in matching retention times and ion abundances of data from different instruments may prevent the differentiation of structurally similar compounds. Also, as a matter of principle, analysts should strive to maximize the power of discrimination available from each dimension of information to provide the maximum affordable confidence in compound identifications. The present study aims to maximize the informative power of the mass spectrometric data.

When coelution occurs between different analytes, chromatographic peaks can be deconvoluted from each other and/or from background ions based on the assumption that the absolute signal intensity of all ions from the same substance correlate perfectly as a function of time.^6,8,15−18^ Once chromatographic peaks are deconvoluted from one another, or background corrected, there are many mathematical approaches to compute spectral similarity or dissimilarity between an extracted spectrum and a reference spectrum.^9,19−22^ Some algorithms use a subset of the spectra, usually of the most abundant peaks, as their searching criteria.^23,24^ However, most approaches struggle to distinguish between compounds with extensive spectral similarity, especially when the rank order of the peaks fluctuates or when any distinct fragments are of low relative abundance.^23,25^

Hertz et al. argued that mass spectral comparisons should include features of the entire spectrum,^23^ so they developed a quantitative measure of similarity—known as the similarity index—that used the two most abundant peaks every 14 Da across the spectrum. McLafferty and Gohlke argued that relatively minor peaks may provide the necessary information for identifications,^26^ and McLafferty and others went on to implement probability-based matching (PBM).^20,27,28^ PBM makes use of “negative information”, such as absent peaks in the unknown spectrum—i.e., the “reverse search”—as well as the peaks present in the spectrum to make an identification. The algorithm is used in Agilent’s software, and the details of these calculations and improvements to this algorithm over the years have been thoroughly documented in the literature.^29,30^

Other popular algorithms used for database search identification during this period are all based on comparing an exemplar or consensus spectrum in a database to the queried spectrum.^31−34^ Examples of common measures of similarity and dissimilarity include the Euclidean distance,^18,19,35^ absolute value distance,^18,19,35^ and the dot-product or cosine similarity algorithm, which compares a query and reference spectrum by calculating the cosine angle between their vector representations.^20,36^ Stein and Scott performed a comparative study of the five most popular algorithms.^20^ Their results showed the dot-product approach to be the best performing algorithm (75% accuracy for Rank 1), followed by Euclidean distance (72%), absolute value distance (68%), PBM (65%), and the Hertz similarity index (64%).^20^ Samokhin et al. found similar lackluster results for different search algorithms from different vendors.^37^

Today, although many new algorithms have appeared for mass spectra from direct analysis in real time-mass spectrometry (DART-MS)^38,39^ and MS/MS data,^36,40−47^ the most widely used mass spectral comparison algorithm for GC–MS data is probably the NIST similarity search algorithm,^20^ which is an adaptation of the simple dot product, *r*, or cosine angle between two spectra^22^
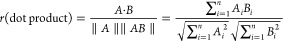
1where *A* and *B* are *n*-dimensional vectors of spectral abundances, *A*_*i*_ is the *i*th element of vector *A*, and *B*_*i*_ is the *i*th element of vector *B*. Typically, *A* and *B* have dimensionality defined by the number of selected *m*/*z* values in the spectra, and they take values of normalized abundances between 0 and 100.

Stein and Scott showed that the ranking order of correct identities could be improved by using an optimized set of arbitrary weight factors (*x*, *y*) to create new weighted variables, *A*_L__(weighted)_, from the peak abundances, [*A*_L_]^*x*^, and corresponding *m*/*z* values, *m*/*z*^*y*^, as demonstrated in eq 2.^20,48^
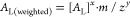
2

Taking the dot product of these weighted variables emphasizes the importance of the larger *m*/*z* values—such as molecular ions—and de-emphasizes the most abundant peaks, which are oftentimes not as spectrally unique as low-abundance fragments. Kim et al. developed a method to determine optimal factors of *x* and *y* in Stein’s algorithm and thereby maximize the accuracy of compound identification using the National Institute of Standards and Technology/Environmental Protection Agency/National Institutes of Health (NIST/EPA/NIH) Mass Spectral Library.^49^ Kim et al. demonstrated that weight factors of *x* = 0.53 and *y* = 1.3 provided an accuracy of 82.83% for #1 ranked correct identities, but they admit that the optimal factors are somewhat arbitrary and are dependent on specific mass spectral libraries, which are often updated.^49^

Stein’s similarity search algorithm is dependent on the relative abundance of peaks in both the unknown and library spectra,^20,49,50^ and although probabilities exist for the ranking accuracy of hundreds of searches, probabilistic measures of accuracy are not available on a compound-specific basis.^50,51^ Therefore, in terms of drug identifications in a forensic setting, NIST match factors have questionable value in helping practitioners meet admissibility criteria described in Federal Rules of Evidence 702, especially for structurally and spectrally related fentanyl analogs.^52^ That said, NIST continues to develop mass spectral comparison tools that help analysts find spectral and structural similarity between questioned spectra and known spectra,^14,38,39,50,52,53^ and these tools will unquestionably assist analysts in identifying rare or novel substances in the future.

Studies by Smith et al. on cocaine diastereomers^54,55^ and Mallette et al. on fentanyl isomers^56,57^ show that the EI-mass spectra between the structurally related analogs are *almost* indistinguishable. However, careful analysis can reveal significant differences in relative ion abundance of a few ratios of ions, such as *m*/*z* 94/96 and *m/z* 152/155 for cocaine diastereomers^54,55^ and *m*/*z* 202/203 and *m/z* 160/216 for 2- versus 3-methylfentanyl.^57^ These studies demonstrate that library-retrieved identification must be supplemented with close expert supervision to enable the discrimination of structurally related analogs.^57^ However, such manual, subjective procedures are not ideal because they are difficult to articulate and defend in a legal setting. Also, such nuanced differences are not transferable to other drugs or analogs.

Smith and McGuffin applied an unequal variance *t*-test on each *m*/*z* value across two spectra (query and library) to determine if spectra were significantly different or not.^58−60^ For phenethylamines, random match probability (RMP) testing provided probabilities on the order of 10^–39^ to 10^–29^, which indicates the very low probability that the characteristic fragmentation patterns occurred by chance. Smith et al. note that the RMP calculations assume that the abundance of each ion in a spectrum is independent, but previous work shows that fragments ions are strongly correlated, with correlation coefficients (*R*) between normalized pairs of ions often exceeding 0.9;^61,62^ hence, the absolute probabilities obtained through RMP are probably overly optimistic. Here, we present an algorithm that demonstrates the ability to make reliable identifications of cocaine, even when the spectra are collected on different instruments and when known positives (KPs) have more obvious differences with the training set than some known negatives (KNs).

Although the expert algorithm for substance identification (EASI) is applied to EI mass spectra of cocaine in this work, the extension to other drugs and to tandem mass spectra of all kinds should be obvious. Given that the validity of EASI relies on the competitive rates of unimolecular fragmentation described by quasi-equilibrium theory/Rice–Ramsperger–Kassel–Marcus (QET/RRKM) theories, as described in the previous manuscript,^62^ EASI should be applicable to any type of mass spectrometer that provides fragments of precursors, whether from EI—as described here—or from electrospray ionization–tandem mass spectrometry (ESI-MS/MS). EASI simply improves the prediction accuracy of fragment ion abundances relative to traditional approaches that use a discrete exemplar or a consensus spectrum as the basis for predictions. Here, we define the predicted abundances of traditional approaches as either those abundances measured in a single reference spectrum, as in a discrete exemplar, or the average/median spectrum from a collection of reference spectra, as in the consensus approach. The consensus approach can take the form of a data array of abundances versus *m*/*z* or abundances with uncertainties versus *m*/*z*.^32,63,64^ Also, whereas the data structure of normalized mass spectra are usually strictly limited to abundances from zero to 100, abundance predictions in EASI are not necessarily limited by the same bounds; known negatives are often so ill-fitting to the cocaine model that abundance predictions less than zero and greater than 100 are quite common. New opportunities for handling such data structures may arise, but existing measures of spectral comparisons handle any negative abundances perfectly well.

## Experimental Methods

As described in our previous manuscript,^62^ general linear modeling (GLM) was used to predict the relative ion abundances for the 20 most abundant fragments in a database of 128 replicates of a cocaine standard that were collected over a 6-month period in an operational crime laboratory. No attempt was made to control the measurement variability beyond typical laboratory procedures. Summary statistics for the measured abundances of different sets of data are provided in Table S1. Each of the 20 most abundant peaks—as defined by the consensus spectrum^32^—was iteratively treated as a dependent variable, and the 19 remaining covariates were added (or removed) in a stepwise manner until there was no significant improvement (i.e., *F* ≥ 0.1) in the amount of variance explained. The GLM resulted in empirical models that contained between three to 12 covariates. The models typically explained more than 90% of the variance of the relative abundance of each dependent ion. The coefficients for each covariate in all 20 models are also provided in Table 2 of our previous manuscript.^62^

Our previous manuscript also suggested that the general linear models built on the training cocaine data from the first crime laboratory (Lab 1) could be extrapolated to make accurate predictions for the 175 cocaine spectra from a different laboratory and 55 cocaine spectra from the NIST archive, which includes spectra collected on a variety of instruments dating back to the 1980s.^62^ This current manuscript provides the details of those claims and outlines different ways to assimilate the 20 predictions within each query spectrum to enable effective binary classification to two groups: “cocaine” and “not cocaine”. In this case, the known negatives include 10 replicate spectra of three cocaine diastereomers, ecgonine methyl ester, heroin, hydromorphone, fentanyl, and methamphetamine. For each future application of EASI to the identification of a particular drug, replicate spectra of each drug would need to be collected and modeled. The obvious computational expenses imply that EASI would only be applied to the top-ranked candidates after a conventional ranking algorithm has already limited the list of likely drug identities.^37,65−67^

The predicted abundances in the two compared approaches (EASI and traditional/consensus) are defined differently. In the consensus approach, the mean spectrum of the training set of 128 spectra from one lab serves as the exemplar spectrum of predicted ion abundances to which all other spectra are compared. This approach represents the status quo in which one assumes there is a “best” or “true” exemplar spectrum of cocaine. In the EASI approach, the relative abundances modeled by the 20 general linear models of cocaine serve as the 20 predicted ion abundances. In EASI, the predicted abundance of a specific fragment (e.g., *m*/*z* 182) in each query spectrum will therefore change depending on the abundance of the other measured fragments in each query spectrum. The beta (β) coefficients in Table 2 of the first manuscript describe the extent to which each variable is used, or not, in each GLM model. Predicted values and the residuals to the measured values of the consensus approach and EASI approach were assessed using various spectral similarity measures, described below, to demonstrate that binary classification using EASI improves the accuracy of cocaine identification relative to conventional methods regardless of the chosen method of spectral matching.

Even though numerous vendors listed various diastereomers of cocaine in their catalogs, no vendors were willing/able to ship any diastereomers. Communications revealed that the isomers had not been recharacterized in recent memory and the vendors could no longer validate the stereochemistry. For these reasons, we could not increase the number of known negative spectra of diastereomers in our database beyond the 10 replicates contained in the NIST archive.

### Mean Absolute Residuals (MAR)

Residuals can be positive or negative, and if the residuals are not skewed, the mean of many unbiased residuals will be zero. There is therefore no value in assessing the mean spectral residual of 20 predications for each spectrum. Instead, we calculated the MAR for the two different approaches—EASI and consensus—using eq 3
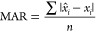
3where *x̂* is the predicted abundance, *x* is the measured abundance, *i* is the *i*th abundance, and *n* is the total number of ions, which in these examples is always the same 20 most abundant ions in the training set of cocaine spectra. Throughout the document, all abundances and residuals are reported relative to each spectrum’s internal base peak at 100%. We prefer the MAR instead of the root mean squared error or predictions (RMSEP) because the MAR does not scale with the number of measurements, so the MAR enables simple comparisons if the number of measurements changes.

### Euclidean Distances

The Euclidean distance, or the square root of the sum of squares of residuals, is another well-known metric to assess the fitness of multivariate predictions.^3,4^ The Euclidean distance (eq 4) is the shortest distance between uncorrelated multidimensional data; in two-dimensional space the Euclidean distance, *d*_Euclid_, is a straight line between two points
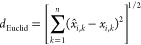
4where *k* is the variable number (1–20 in this work), *x̂*_*i*_ is the predicted abundance, and *x*_*i*_ is the measured abundance for the *i*th spectrum. Given that the residuals in EASI are not significantly correlated among the 20 models for cocaine,^62^ the Euclidean distance is a valid approach to assess the residuals. However, the strong correlation between raw and normalized ion abundances makes the Euclidean distance unsuitable for assessing distances on the original peak heights.

### Dot Products and NIST Scores

Within each query spectrum, each of the 20 measured abundances were compared to the 20 predicted abundances using either GLM models in the EASI approach or the mean abundances of the training set in the consensus spectrum approach. Dot products were calculated according to eq 1, and NIST scores were calculated according to eqs 1 and 2 using Stein’s original weighting factors of *x* = 0.6 and *y* = 3 and a scaling factor of 999.^20^

### Mahalanobis Distances

The Mahalanobis distance can be thought of as the Euclidean distance in multidimensional space after a whitening transformation via the covariance matrix to remove the covariance between the variables.^68^ The Mahalanobis distances can be calculated directly on the normalized spectra, and no separate linear modeling is necessary. Given the extensive covariance between relative ion abundances in replicate spectra, it seemed reasonable to assess the Mahalanobis distance^69^ of every spectrum in the database of 1019 spectra to the multidimensional space defined by the variance of the 128 training spectra of cocaine from Lab 1.^70^ The Mahalanobis distance *d*_Mahal_ to the central mean of a training set is defined as
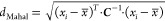
5where *x*_*i*_ is an object vector, *x̅* is the arithmetic mean vector, ***C*** is the sample covariance matrix (eq 5), and *T* is the transpose operator. The distances can be interpreted as being equivalent to the number of standard deviations away from the mean in multidimensional space, and they can either be compared to statistically relevant distances, such as using the Hotelling’s *T*^2^ test,^70^ or simply relative to one another in receiver operating characteristic (ROC) curves,^71−73^ as is done here. Strictly speaking, the Mahalanobis distance is designed to work in well-calibrated situations in which the training set includes all the expected sources and magnitudes of variance as the queried samples.^68^ Therefore, one notable difference between GLM employed in EASI and the Mahalanobis distance is that GLM can—in theory—be extrapolated to model behavior that is outside the measured variance of the training set, as demonstrated in part 1 of this manuscript.^62^ Therefore, known-positive query spectra from outside the training set—such as those collected on different instruments or using different conditions—that are significantly different from the training set with respect to their Mahalanobis distances should still be accurately predicted and not erroneously rejected by EASI’s GLM.

### Receiver Operating Characteristic (ROC) Curves

A ROC curve is a graphical visualization of the true positive rate (TPR), or sensitivity, versus the true negative rate (TNR), or 1-specificity.^71,72^ ROC curves allow users to determine the effectiveness of similarity and dissimilarity metrics as binary classifiers, and they can be used to evaluate binary decision algorithms like “yes” and “no” to a chemical identification.^74,75^ Using the measures of mass spectral similarity and dissimilarity described above as continuous variables, the number of true positives (TPs), true negatives (TNs), false positives (FPs), and false negatives (FNs) were assessed at a variety of threshold values for every spectrum in the database. A plot of TPR vs 1-TNR (the false positive rate) allows users to see the trade-off between the TPs and TNs when various thresholds are chosen for binary decisions. In addition, one can visualize a confusion matrix of the number of TPs, TNs, FPs, and FNs at each threshold value.
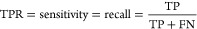
6
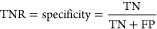
7
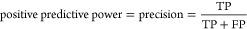
8

We also report the area under the curve (AUC) for the ROC curves, which is a well-accepted measure of a test’s general discriminating power.^5,8,37,40,71,76−78^ The AUC informs a user how well a model correctly identifies a substance over the range of decision points. An AUC of 1.0 is the maximum value and indicates a perfect test, or errorless identification, in which all known negatives have measures of dissimilarity that are larger than all known positives (or in which all known negatives have measures of spectral similarity that are smaller than all the known positives). An AUC of 0.5 indicates a 50% chance of correct classification, which is not better than a random classifier, so it holds no discriminatory value. Of course, AUCs closest to 1 are most desirable. ROC curves were assessed using various measures of spectral comparisons, including the dot products, NIST scores, MARs, Euclidean distances, and Mahalanobis distances. Precision–recall curves are also presented to better understand the positive predictive power of the unbalanced data set.^79^ Precision–recall curves that cross the boundary condition at (1,1) are perfect classifiers.

## Results and Discussion

**Figure 1:**
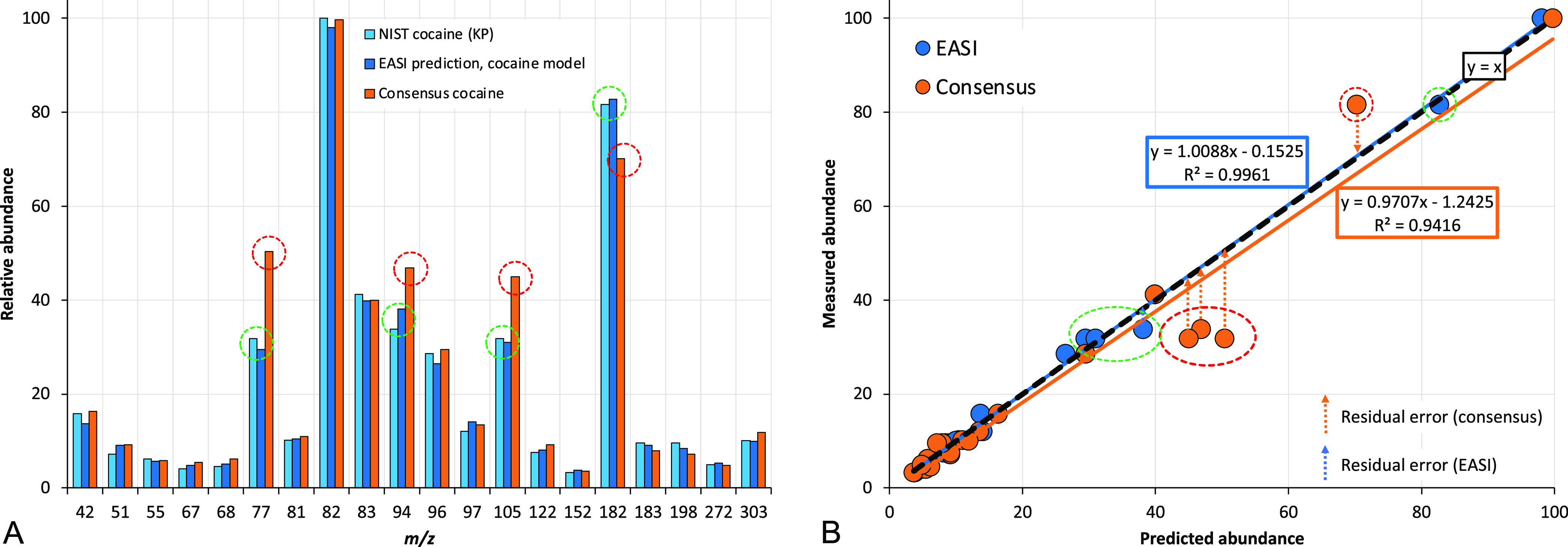
(A) Bar chart to show one NIST validation spectrum of cocaine (a known positive [KP]), relative to both the consensus spectrum abundances of cocaine from Lab 1 and EASI-predicted abundances based on the same training set. (B) Scatter plot of the same data to show the origins of the residual errors of the predictions. Red ovals highlight large residual differences, which are unfavorable for a known positive, and green ovals indicate improved predictions. The dashed black line in B shows *y* = *x* for errorless predictions.

Our previous manuscript described the kinetic basis for using GLM to model the ion abundances of ions in replicate spectra.^62^ As a proof-of-concept, we are continuing the description of the proposed algorithm to the same database of cocaine spectra.^61,62^ Example predictions from two types of models are presented in Figure 1.

In light blue are the measured values from one of the validation spectra of cocaine from the NIST archive. This spectrum was collected on a different instrument, in a different decade, than those in the training set. In orange are the consensus (mean) abundances of the training set. In conventional statistics, the mean values of the training set would normally serve as the best estimates for the population mean or “true” values because *x̅* → μ. For at least the three fragments circled in green, *m*/*z* 77, 94 and 105, the abundances in the NIST cocaine spectrum are at least 10% smaller (relative to the base peak) than the consensus cocaine spectrum, which are circled in red. In contrast, the abundance of *m*/*z* 182 in the NIST spectrum is around 10% larger than in the consensus spectrum. These residual errors in abundance predictions of ∼10% or more are readily observable in the bivariate plots of measured vs predicted abundances in Figure 1B. For reference, errorless predictions are denoted by the dashed black line *y* = *x*. Even though this NIST cocaine spectrum has obvious differences from the consensus cocaine spectrum from Lab 1, the 20 GLM models used in the EASI approach make better predictions than the consensus spectrum approach. Such improvements in predictions are only possible because the deviations between the training set and the NIST spectrum are systematic in nature and not random. These systematic differences are explained mathematically by QET/RRKM theories, as demonstrated in our previous manuscript.^62^

Figure 1B shows that for the 20 predicted ion abundances within the spectrum it is possible to derive 20 residual errors or one Pearson product–moment correlation (PPMC) value (*R*). As described in the Experimental Methods, the 20 residual errors in each predicted spectrum can then be assimilated into a single measure of spectral dissimilarity in a variety of ways. One note of caution regarding the interpretation of PPMC values is that it is theoretically possible for all the predicted values to differ from all the measured values yet still provide a PPMC of 1. Such a case could happen, for example, if all the predictions had a constant proportional error. For this reason, dot products are used here instead of PPMC values. Still, as a rule of thumb, coefficients of determination, *R*^2^ values closer to 1 are generally indicative of more accurate predictions and can be interpreted as increasing the confidence in the correct identity for the model.

In Figure 1B, the consensus cocaine spectrum provides an *R*^*2*^ value of 0.9416 relative to the query cocaine spectrum from NIST, but the GLM-predicted abundances in EASI provide an *R*^*2*^ value of 0.9961, which is a better fit. The MAR and Euclidean distances for the consensus spectrum were 3.74% and 29.16%, respectively, but only 1.26% and 7.19%, respectively, for the GLM-based modeling in EASI, which in both cases is at least three times more accurate than the consensus approach. For all the KPs of cocaine, the accuracy of predictions was consistently better for EASI than the consensus approach.

**Figure 2:**
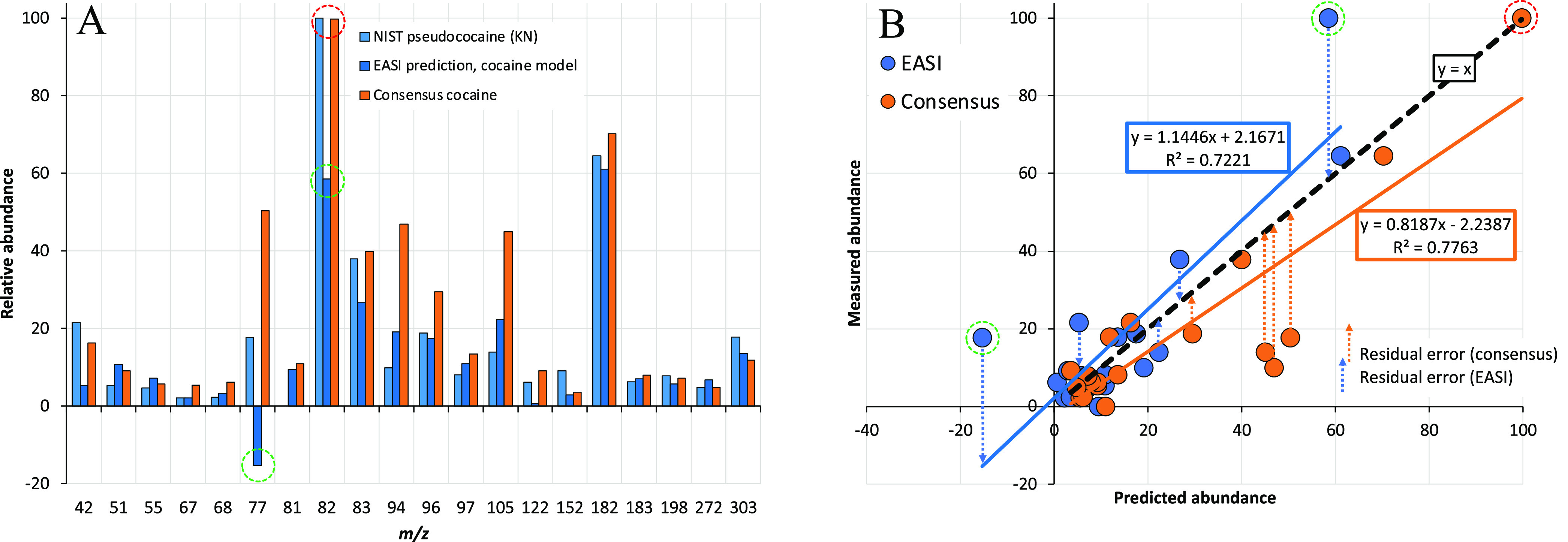
(A) Bar chart to show one NIST validation spectrum of pseudococaine, a known negative (KN), relative to (i) the consensus spectrum of cocaine from Lab 1 and (ii) the EASI-predicted abundances. (B) Scatter plot of the same data to show the origins of the PPMC values and residual errors of the predictions. Red ovals highlight small residual differences, which are unfavorable for a known negative, and green ovals indicate larger residual errors, which are desirable in this case. The dashed black line in panel B shows *y* = *x* for errorless predictions.

Figure 2 shows the same approach when a KN pseudococaine spectrum is carried through the cocaine GLM model. In this case, the spectrum was also from the NIST archive and therefore on a different instrument. Both the consensus and EASI approaches produce larger residual errors than the known positive cocaine spectrum in Figure 1, which is a desirable property of KNs in a discriminating algorithm. Some ions of pseudococaine—like *m*/*z* 97, 105, and 303—have abundances that coincidentally are accurately predicted by the cocaine GLM models, whereas other fragments—like *m*/*z* 77 and 82—are poorly predicted by the cocaine GLM models and have residual errors as large 33% and 41%, respectively.

The GLM-predicted abundances for the cocaine model and the consensus cocaine spectrum both share reasonable correlation with the KN NIST pseudococaine spectrum, with *R*^*2*^ values of 0.7221 and 0.7763, respectively. Like the GLM-predicted abundances, the consensus spectrum of cocaine differs from the pseudococaine spectrum most significantly at *m*/*z* 77, 94, and 105. The abundance at *m*/*z* 94 is a well-documented point of differentiation between the two stereoisomers.^54,55,80^

For the same spectra in Figure 2, the MAR and Euclidean distances of the consensus spectrum approach were 8.50% and 62.04%, respectively. The same measures of dissimilarity using the GLM models in EASI were 8.33% and 60.05%, respectively. In this case, the pseudococaine spectrum is almost equally dissimilar using either the consensus or EASI approach. In general, most of the KNs in the database provided similar measures of spectral similarity and dissimilarity using either the EASI or consensus approach, so both approaches are equally ineffective at predicting ion abundances of the stereoisomers of cocaine. Summary statistics of the MARs for the different spectral sets are provided in Table 1. Additional summary tables for other measures of spectral comparisons—dot products, NIST scores, Euclidean distances, mean absolute residuals, and Mahalanobis distances—are provided in Supplemental Table S2.

**Table 1:** Summary of Mean Absolute Residuals (MARs) between Measured Ion Abundances and the Two Different Models—Consensus Model and EASI—for a Variety of KPs and KNs

		Mean absolute residuals (MARs) (% relative to base peak)			
		Standard consensus model		EASI	
Spectral set		Mean of set	Range of set^a^	Mean of set	Range of set^a^
Known positives (KPs)	Lab #1 cocaine; training set (*N* = 128 spectra)	3.10	1.07–8.17	0.69	0.18–1.81
	Lab #2 cocaine; prediction set (*N* = 120 spectra)	6.36	2.86–12.25	1.43	0.74–2.80
	NIST cocaine; validation set (*N* = 55 spectra)	5.49	2.15–15.07	1.95	0.49–5.18
Known negatives (KNs)	Five drugs from Laboratories 1 and 2 (*N* = 706 spectra)	21.15	14.93–24.37	10.39	7.28–24.10
	NIST allococaine (*N* = 1 spectrum)	8.56		5.47	
	NIST pseudococaine (*N* = 7 spectra)	6.30	5.75–11.25	5.07	2.07^c^–7.38
	NIST pseudoallococaine (*N* = 2 spectra)	6.8	5.73^b^ −6.87	4.52	4.48–4.55

a MAR is defined in eq 3. If the MAR for any KP exceeds that of any KN, errorless predictions are not possible.

b A threshold value of 5.72 for the consensus model has 0 FPs and a total of 83 FNs.

c A threshold value of 2.06 for EASI has 0 FPs and a total of 36 FNs.

For the KPs from Laboratories 1 and 2, the MARs were, on average, 4.4 times smaller using EASI than using the consensus approach. The mean MAR of the NIST archive KPs was 2.8 times smaller using EASI than the consensus approach. The EASI models for cocaine also made more accurate predictions for most of the KNs, but the reduction in MARs for the KNs was smaller than for the KPs. The net result is that EASI modeling increased the difference between the MARs of KN spectra and KP spectra because the abundance predictions are vastly more accurate than the consensus approach for all the KPs of cocaine than for the KNs. The reason that known negatives with dissimilar fragmentation patterns to cocaine can have improved MARs or NIST scores for EASI relative to the consensus approach is that when the measured and EASI-predicted abundances are both near zero for a certain peak they inadvertently provide a near-zero residual error. Therefore, spectrally distinct compounds may have measures of spectral similarity that appear to improve from the consensus to the EASI approach. Future applications of EASI should therefore either be limited to spectrally similar compounds or should incorporate a penalty when many of the expected abundances measure zero or near zero.

**Figure 3:**
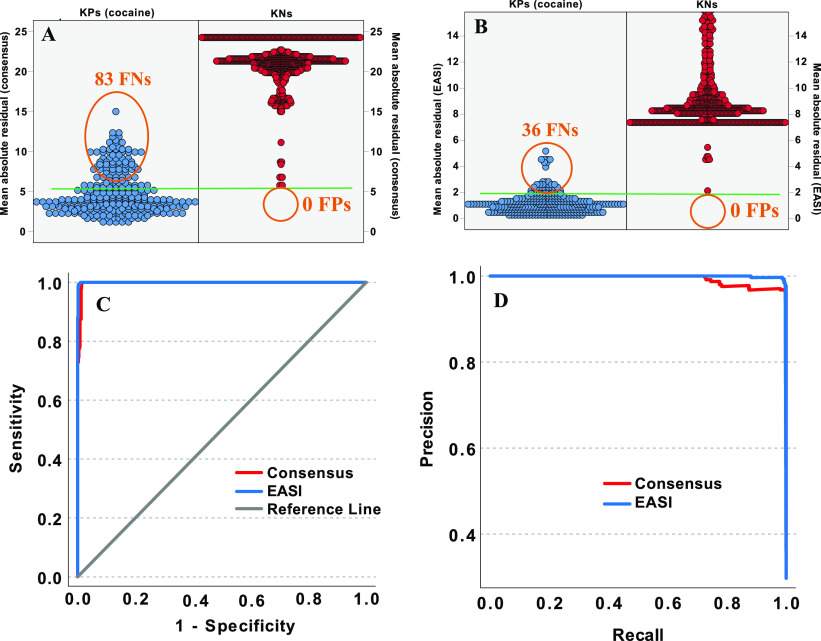
Frequency distribution plots for mean absolute residuals (MARs) for the consensus (A) and EASI (B) approaches to spectral predictions. (C) Receiver–operator characteristic (ROC) curves for the two models. (D) Precision–recall curve for the two models.

To visualize and better understand the distributions of spectral similarity scores for the different models, frequency distribution curves of the MARs for the two models are provided in Figure 3. All the KP spectra of cocaine are in blue and all the KN spectra, including the cocaine diastereomers and ecgonine methyl ester, are in red. Figure 3A shows that there are several KN spectra with MARs between 5 and 10% that fall within the range of MARs of the KP cocaine spectra. Figure 3B shows that the distributions of MARs using EASI overlap less, so binary classification results in fewer FPs and FNs. At a threshold of ∼2% MAR, EASI results in no false positives and 36 false negatives, most of which are external validation cocaine spectra from the NIST archive from unknown instruments. In both figures, the overlap in MARs between KPs and KNs results from the cocaine diastereomers. In the consensus approach, one cocaine spectrum had a MAR that exceeded those of several spectra of ecgonine methyl ester collected on the same instrument.

The areas under the ROC curves in Figure 3C are 0.9998 for EASI and 0.9974 for the consensus approach. Although both classifiers can easily distinguish most cocaine spectra from most known negatives, EASI obviously provides better discrimination in cases where the cocaine spectra contain more variance from the training set, such as those in the validation set that were collected on different instruments. Figure 3D shows the precision–recall curve for the same data, which again shows the improvements in precision for EASI relative to the consensus approach.

Table 2 summarizes the NIST scores between the measured ion abundances for the consensus approach and EASI. In both approaches, some of the NIST scores for KNs of pseudococaine exceed the scores of some KP cocaine spectra. The spectral similarity of the diastereomers to cocaine therefore causes an overlap in the distribution of NIST scores for KNs and KPs, meaning that errorless classifications are not possible.

**Table 2:** Summary of NIST Scores between Measured Ion Abundances and Two Different Models of Exemplars—consensus Model and EASI—for a Variety of KPs and KNs

		NIST scores			
		Standard consensus model		EASI	
Spectral set		Mean	Range of set	Mean	Range of set
Known positives (KPs)	Lab #1 cocaine; training set (*N* = 128 spectra)	995.9	980.7–998.9	998.4	992.6–999.0
	Lab #2 cocaine; prediction set (*N* = 120 spectra)	991.2	976.7–998.6	998.1	984.0–999.0
	NIST cocaine; validation set (*N* = 55 spectra)	993.9	979.2–998.8	992.4	885.3–998.9
Known negatives (KNs)	Five drugs from Labs #1 and #2 (*N* = 706 spectra)	298.6	67.8–499.1	379.2	8.51–913.9
	NIST allococaine (*N* = 1 spectrum)	990.9^a^		973.0	
	NIST pseudococaine (*N* = 7 spectra)	992.2	988.6–**996.8**^a^	978.0	959.0–**996.0**^b^
	NIST pseudoallococaine (*N* = 2 spectra)	992.5	988.7–996.2	960.2	975.8–994.7

a A threshold NIST score of 996.9 for the consensus approach yields 0 FPs and 179 FNs.

b A threshold NIST score of 996.1 for EASI yields 0 FPs and 28 FNs.

If we assume the highly conservative threshold of zero false positive identifications, the consensus approach using NIST scores yields 179 FNs (59% false negative rate) at a threshold of 996.9. In contrast, the EASI approach using NIST scores yields zero FPs and 28 FNs (9.2% false negative rate) at a threshold of 996.1. This head-to-head comparison of the two approaches shows that EASI provides better abundance predictions for KPs collected on different instruments than the consensus approach. Binary classification of cocaine using NIST scores of EASI abundances provided the lower error rate of the two approaches, with a combined accuracy of 97.3% for all 1019 identifications. NIST scores using the traditional consensus cocaine spectrum approach provided an overall accuracy of 82.4%, which masks the fact that more than half of the cocaine spectra were incorrectly classified.

**Figure 4:**
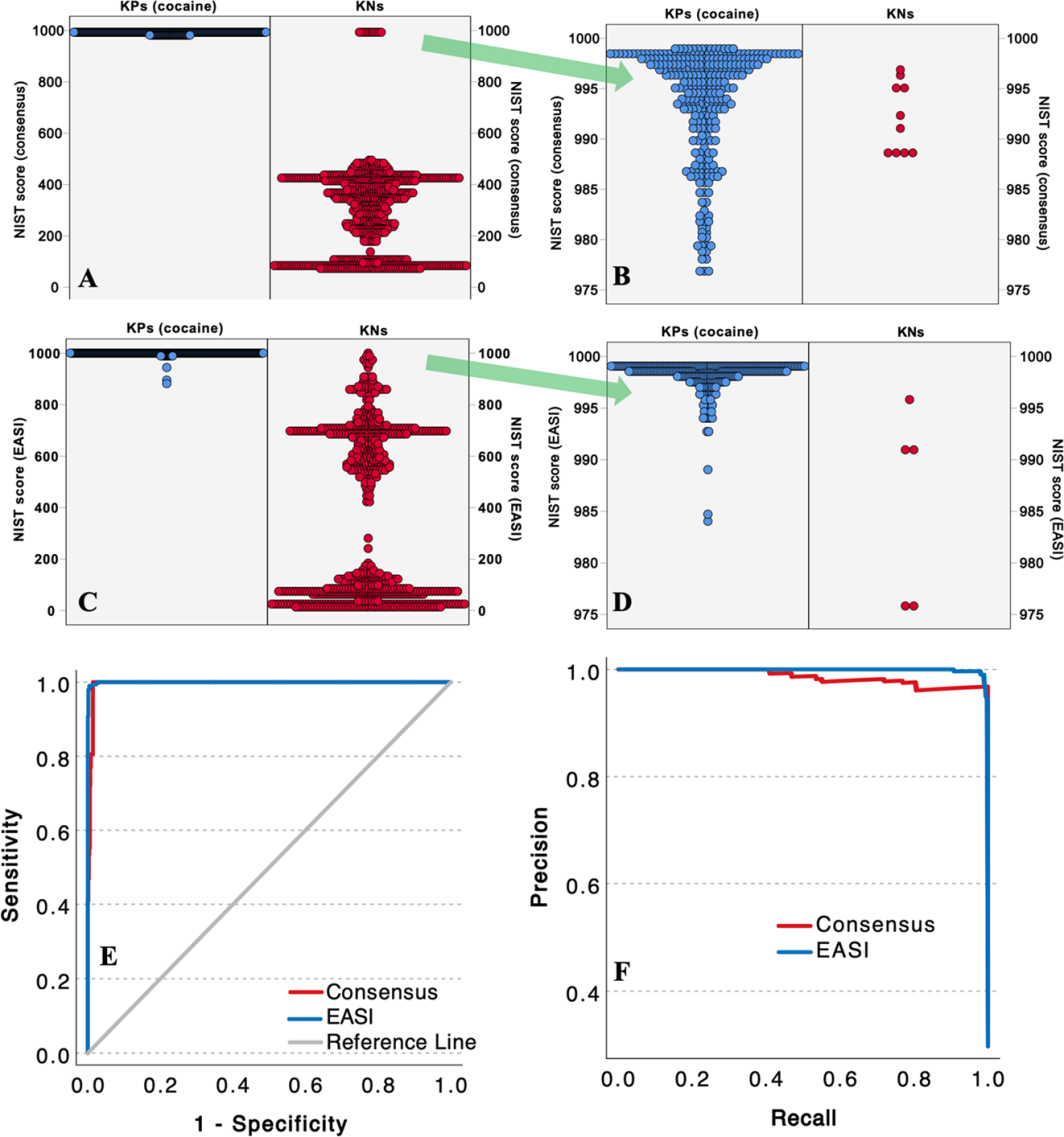
Population plots for NIST scores for the consensus (A, B) and EASI (C, D) approaches to spectral predictions. (E) Receiver–operator characteristic (ROC) curves for the two models. (F) Precision–recall curve for the two models.

Population plots for the NIST Scores for the two models are provided in Figure 4. All the KP spectra of cocaine are in blue and all the KN spectra, including the cocaine diastereomers and ecgonine methyl ester are in red. Figure 4A,B shows that, for the consensus approach, there are several KN spectra with NIST scores between 985 and 996 and dozens of KP cocaine spectra with NIST scores between 975 and 990, which mostly include the validation spectra from different instruments. The population of known positives and negatives overlap severely in the consensus approach. In contrast, the distribution of NIST scores in Figure 4C,D using EASI is more condensed, and there are only a handful of KP spectra from the validation set with NIST scores less than 990. The areas under the ROC curves (AUC) in Figure 4E are 0.9996 for EASI and 0.9954 for the consensus approach. Although both classifiers can easily distinguish most cocaine spectra from KNs that are not diastereomers, EASI obviously provides better discrimination between cocaine and its diastereomers. Figure 4F shows the precision–recall curve for the same data, which again shows the improvements in precision for EASI relative to the consensus approach.

**Figure 5:**
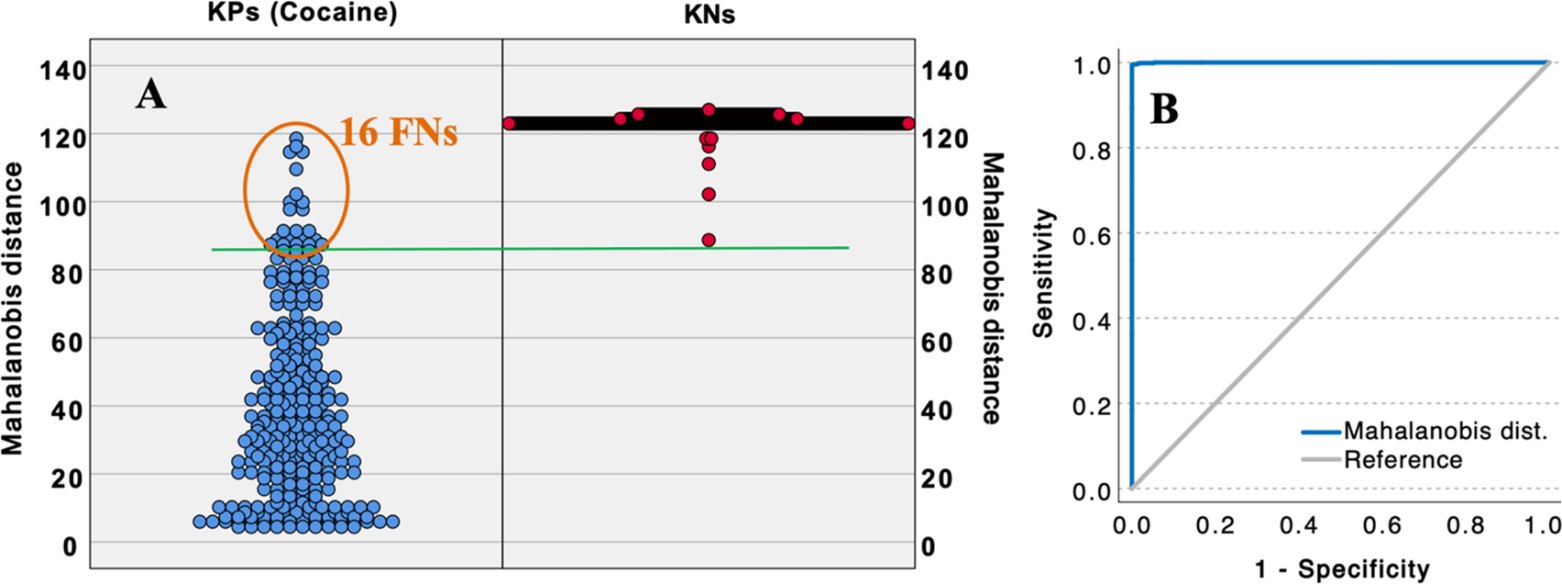
Population plots for the Mahalanobis distance to the training set of 128 cocaine spectra for KPs and KNs (A) and a corresponding receiver-operator characteristic (ROC) curve (B).

Table 3 summarizes the TPs, TNs, FPs and FNs for the different models and different methods of spectral comparison. The error rates are somewhat biased by the inclusion of the training set with the test and validation sets, but EASI and the consensus approach were treated similarly with the different data sets, so the relative comparisons of EASI and the consensus approach are still valid. Within each method of spectral comparison, EASI results in more TPs and greater overall accuracy than the consensus approach. Using the Mahalanobis distances (to the training set) as a binary classifier provides more TPs than any consensus approach or EASI approach when the threshold is set at zero FPs. The population plot and ROC curve in Figure 5 help visualize the distributions and classification performance. At the threshold of zero FPs, the Mahalanobis distance only predicted 16 FNs, for a FN rate of 5.2%. The AUC for Mahalanobis distance in Figure 5B is 0.9998, which is more effective than the consensus approach, but approximately equal to EASI. Like all the metrics studied using EASI, Mahalanobis distance struggles to differentiate several of the diastereomer spectra from a few of the external validators of cocaine.

**Table 3:** Summary of Binary Classification Figures of Merit for Various Measures of Spectral Similarity for EASI and the Consensus Model for Cocaine Identification in the Absence of Retention Time Information Relative to the Cocaine Training Set^a^

	Dot product		NIST score		Euclidian distance		MAR		
Model	Consensus	EASI	Consensus	EASI	Consensus	EASI	Consensus	EASI	Mahalanobis distance
Threshold	0.968	0.997	996.8	996.0	41.66	12.31	5.73	2.07	88.19
TPs	214	250	124	275	219	247	220	267	287
TNs	716	716	716	716	716	716	716	716	716
FPs	0	0	0	0	0	0	0	0	0
FNs	89	53	179	28	84	56	83	36	16
Sum	1019	1019	1019	1019	1019	1019	1019	1019	1019
FPR =	0.0	0.0	0.0	0.0	0.0	0.0	0.0	0.0	0.0
**TPR =**	**70.0%**	**81.8%**	**51.8%**	**96.4%**	**72.3%**	**81.5%**	**72.6%**	**88.1%**	**94.7%**
**TNR =**	**100.0%**	**100.0%**	**100.0%**	**100.0%**	**100.0%**	**100.0%**	**100.0%**	**100.0%**	**100.0%**
**Accuracy**	**91.3%**	**94.8%**	**82.4%**	**97.3%**	**91.8%**	**94.5%**	**91.9%**	**96.5%**	**98.4%**^a^

a Only the 20 most abundant peaks in a spectrum are compared in each model, and the threshold is set to exclude FPs. Mahalanobis distances are calculated for every spectrum relative the cocaine training set from Lab 1.

Although confusion matrices *can* be used to calculate likelihood ratios (LR) or log likelihood ratios (LLR) as a measure of the informative power of the methods,^58,76,81^ the LR will be unique for each spectrum because spectra that are further from the threshold will be more confidently assigned to the correct group. Spectra closer to the threshold will be less confidently and less accurately identified. Greater than 95% of the KPs can be confidently and correctly identified as cocaine without any FPs. Conversion of spectral comparison scores to LRs would require kernel density estimations and computing the extent of overlap of the continuous distributions at different thresholds.^76,82−85^ Such calculations are beyond the scope of the present proof-of-concept, but some example likelihood ratios for the overall performance of the different metrics are provided in Table S3 as an example. The likelihood ratios are severely underestimated here because of the limited number of KNs in the database. Translating error rates and likelihood ratios to casework will require significantly more validation than provided here, including the incorporation of casework sample that are unequivocally found to be known negatives and known positives. Future validation studies should include mass spectra derived from casework samples, which should include the variety of concentrations, cutting agents, and matrix effects expected in practice.

Incidentally, one could also perform a Chi-squared test of Mahalanobis distances as a test for spectral outliers relative to the training set of KP cocaine spectra. A Chi-squared test using 19 degrees of freedom (a very conservative estimate) and *p* = 0.05 provides a critical value of 30.1, which would result in 170 of the cocaine spectra labeled as outliers relative to the training set. The arbitrary threshold Mahalanobis distance of 88.19 results in zero FNs and only 16 FPs for a total accuracy of 98.4%.

The error rates and accuracies in Tables 3 and S3 for the 5 different measures of spectral comparisons reveal some important outcomes, which may be generalizable to other systems: (1) the Mahalanobis distance is an effective metric for binary classification, (2) EASI outperforms the consensus approach regardless of the spectral comparison approach, and (3) EASI can be employed effectively with a wide variety of spectral similarity measures. Indeed, EASI is not necessarily a new way to compute spectral comparisons, it is a new way to assess how well the abundances within a spectrum fit the pattern of behavior (as defined by general linear models) observed for known positives in a training set. The unique feature of the general linear modeling employed here is that, like any linear equation, they can be extrapolated beyond the range of measured values of the training set to make accurate abundance predictions for spectra of known positives from different laboratories that are apparently spectrally distinct but follow the expected linear behavior. Users can use their preferred method of choice to compare EASI-predicted abundances to the measured abundances in a query spectrum.

The comparisons provided above all make use of the 20 most abundant fragment ions in their comparisons. No attempt was made to optimize or supervise the use of specific variables to improve the identification performance. The models were built naïvely with respect to all negatives. However, as mentioned in the introduction, the abundance ratios such as *m*/*z* 94/96 and 152/155 are well-documented points of differentiation between the two stereoisomers,^54,55,80^ so emphasizing these variables in an identification algorithm ought to improve the selectivity between cocaine and its diastereomers. Using the residual errors (ε) between EASI predictions and measured abundances as input variables into binary logistic regression analysis, stepwise addition of the 20 variables resulted in a simple model that resolved all the cocaine spectra from all the diastereomers with no errors. The equation
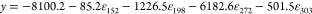
9yields *y* < 0.5 for all 303 spectra of cocaine and *y* > 0.5 for the ten replicate spectra of the three diastereomers, pseudococaine, allococaine, and pseudoallococaine, and therefore enables errorless identification of cocaine using *y* = 0.5 as the threshold. One must be cautious about extrapolating probabilities from one study (i.e., this study) to casework, but the goal of this current project is to demonstrate a framework for maximizing the information in mass spectra rather than providing one specific solution for one specific drug. Cocaine and its diastereomers were used here because their discrimination is such a difficult case. Still, the binary logistical regression equation in eq 9 only requires 4 modeled abundances to distinguish cocaine from its diastereomers, and each of those abundances is easily computed from 4 linear equations with fewer than ten terms each. As a reminder, the GLM equations were built on 128 spectra from Lab 1 and tested against 120 cocaine spectra from a different laboratory and 55 cocaine spectra from the NIST archive, which includes spectra collected on a variety of instruments dating back to the 1980s.^62^ The original GLMs were also naïve with respect to any known negatives.

Regarding the applicability of general linear modeling to other substances and to tandem mass spectra, we have already tested EASI on more than a dozen substances using EI-MS data and more than a dozen substances using replicate tandem mass spectra from a linear ion trap mass spectrometer and a quadrupole-time-of-flight (Q-TOF) mass spectrometer. In all cases, EASI provided superior abundance predictions and more accurate identifications relative to the consensus approach.

Regarding the number of replicate spectra required to build an effective model, the answer depends on many factors, including the number of variables modeled, the spectral similarity of the nearest known negative(s), and the variance observed in the training set relative to the variance in the entire population of known positives. In general, we have found that the accuracy of spectral predictions scales approximately linearly with the square root of the number of replicate spectra in the training set, and about 30 spectra are typically sufficient to provide notably better discriminating power than the consensus approach. As with any algorithm, prediction accuracies will be highest when the overall variance in replicate spectra is minimized and when the training set incorporates all the variance of the population. In real applications, where those ideals are not achievable, EASI provides a unique approach to extrapolate beyond a training set and make interlaboratory spectral comparisons that are more reliable than existing approaches.

At present, it takes several hours to manually process the data and validate the various general linear models for a substance. In the future, such model generation and validation could be automated through scripting to complete in seconds. However, once linear models are developed using a robust training set for a substance, they should not need to be recalculated again. Therefore, the coefficients for a substance (such as those provided in Table 2 of our previous manuscript), should be valid in perpetuity. Once developed, applying *n* linear models to a query spectrum could be readily accomplished in an Excel spreadsheet, where one could also compute measures of spectral similarity and corresponding probabilities of compound identification in a fraction of a second.

## Conclusion

The newly developed general linear modeling in EASI improves upon the exemplar approach to substance identification. EASI considers the fact that normalized ion abundances in a mass spectrum are not independently variable but correlate or anticorrelate with one another in approximately linear fashions according to QET/RRKM theories. The multivariate models typically explain more than 90% of the variance in replicate spectra, and the models can be used to predict ion abundances with residuals that are typically 4 times smaller than the consensus approach, which uses the mean spectrum of all the known positives in the database as the true or predicted abundances. Five different measures of spectral similarity and dissimilarity were compared between EASI and the consensus approach, and in every case EASI provided fewer FNs when the threshold was set to zero or one FP. ROC curves and precision–recall curves showed that binary classification using EASI was superior to the consensus approach for every metric.

Several models using EASI, including mean absolute residuals and NIST scores, provided FN rates of less than 5% with no FPs. Supervised classification of the residuals from EASI predictions were built using binary logistic regression. The binary logistical equation for resolving cocaine from its diastereomers only requires the residuals from four modeled ion abundances to provide errorless identifications of the tested spectra. These findings include more than 175 test and validation spectra of cocaine collected in more than a dozen laboratories, over more than three decades, on unknown instruments, without retention time information, and with 10 spectra of three diastereomers that differ only in the stereochemistry of two chiral centers. Ongoing work demonstrates that EASI is equally well suited to intra- and interlaboratory comparisons of tandem mass spectra from ESI-MS/MS and DART-MS/MS instruments.

## Supporting Information Available

Summary statistics for 20 ion abundances of different groups of compounds (Table S1); summary of spectral similarity measures for consensus and EASI identification of cocaine (Table S2); and (3) summary of binary classification figures of merit for consensus and EASI identification of cocaine (Table S3) (PDF)
