# Supplementary material for: Expert Algorithm for Substance Identification Using Mass Spectrometry: Application to the Identification of Cocaine on Different Instruments Using Binary Classification Models
Source: J Am Soc Mass Spectrom. 2023 May 31;34(7):1235–47. doi: 10.1021/jasms.3c00090 (PMC10326919; doi:10.1021/jasms.3c00090)
Supplement: Supplementary file 1 — js3c00090_si_001.pdf [file js3c00090_si_001.pdf]

Supporting Information

Expert Algorithm for Substance Identification (EASI) using Mass Spectrometry: Application to the Identification of Cocaine on Different Instruments using Binary Classification Models

Samantha A. Mehnert,[a,b,#] J. Tyler Davidson,[a,$] Alexandra Adeoye,[a] Brandon D. Lowe,[b] Emily A. Ruiz,[b] Jacob R. King,[b] Glen P. Jackson[a,b*]

[a]*Department of Forensic and Investigative Science, West Virginia University, Morgantown, WV 26506, USA.*

[b]*C. Eugene Bennett Department of Chemistry, West Virginia University, Morgantown, WV 26506, USA.*

[#]*Current Address: Department of Chemistry, Purdue University, West Lafayette, IN 47907, USA.*
[$]*Current Address: Department of Forensic Science, Sam Houston State University, Huntsville, TX, 77340, USA.*

[*]glen.jackson@mail.wvu.edu; *304-293-9236*

Table S1. Summary statistics for 20 ion abundances of different groups of compounds.

Report

Set#_4		mz42	mz51	mz55	mz67	mz68	mz77	mz81	mz82	mz83	mz94	mz96	mz97	mz105	mz122	mz152	mz182	mz183	mz198	mz272	mz303
Cocaine training	Mean	16.46	9.31	5.80	5.52	6.21	50.64	10.94	99.69	39.90	47.05	29.50	13.54	45.21	9.21	3.62	70.60	7.98	7.19	4.88	12.02
	N	128	128	128	128	128	128	128	128	128	128	128	128	128	128	128	128	128	128	128	128
	Std. Deviation	7.11	3.78	1.67	.86	.91	12.07	.53	2.42	1.77	5.91	2.70	1.67	8.46	1.10	.54	14.59	1.70	1.41	1.81	5.48
Cocaine validation	Mean	16.50	8.19	5.84	4.58	4.79	31.17	10.15	91.01	35.42	34.12	25.16	11.62	30.70	8.81	4.23	88.43	10.10	10.59	8.38	21.34
	N	175	175	175	175	175	175	175	175	175	175	175	175	175	175	175	175	175	175	175	175
	Std. Deviation	4.80	2.11	1.48	1.16	.91	5.04	1.91	12.65	5.63	5.19	3.40	1.87	3.74	1.25	.87	15.24	1.57	2.14	3.14	9.70
Negatives; diasteriomers	Mean	14.81	5.09	4.90	2.93	3.84	23.28	6.29	96.90	37.31	18.38	22.10	9.27	21.01	7.12	9.86	72.70	7.53	9.59	4.21	12.85
	N	10	10	10	10	10	10	10	10	10	10	10	10	10	10	10	10	10	10	10	10
	Std. Deviation	7.43	2.87	.85	1.78	.93	6.52	4.56	8.26	4.90	7.15	3.22	1.55	5.41	1.19	4.37	16.06	1.69	3.19	1.39	4.34
Negatives; other	Mean	24.45	3.67	7.21	2.92	4.49	11.28	6.40	17.57	8.26	8.69	22.50	5.25	8.11	1.48	4.12	2.48	1.33	3.31	.01	.00
	N	706	706	706	706	706	706	706	706	706	706	706	706	706	706	706	706	706	706	706	706
	Std. Deviation	22.23	3.35	8.24	2.62	5.33	10.05	7.97	29.79	16.34	8.18	25.59	10.03	7.68	1.99	5.35	2.66	1.59	4.98	.05	.00
Total	Mean	21.99	5.17	6.77	3.53	4.75	19.75	7.61	41.28	17.18	17.97	23.83	7.43	16.78	3.76	4.14	26.49	3.73	5.11	2.10	5.30
	N	1019	1019	1019	1019	1019	1019	1019	1019	1019	1019	1019	1019	1019	1019	1019	1019	1019	1019	1019	1019
	Std. Deviation	19.15	3.96	6.94	2.46	4.50	16.89	6.95	43.79	19.29	16.33	21.50	9.04	15.48	3.87	4.53	37.39	3.99	5.14	3.60	9.48

Table S2. Summary of spectral measures of similarity and dissimilarity for the consensus approach and EASI approach to predicting ion abundances in different data sets.

	Metric	Dot product		NIST score*		Euclidean distance		MAR		Mahalanobis distance
**Set**	**Statistic**	**EASI**	**Consensus**	**EASI**	**Consensus**	**EASI**	**Consensus**	**EASI**	**Consensus**	
**Cocaine training**	**Mean**	0.99954	0.99267	998.36	995.88	4.66	22.70	0.69	3.09	19.8
	**N**	128	128	128	128	128	128	128	128	128
	**Std. Deviation**	0.00051	0.00686	1.04	3.46	2.63	8.61	0.36	1.19	16.2
	**Minimum**	0.99690	0.94640	992.62	980.72	1.08	7.62	0.18	1.07	3.9
	**Maximum**	1.00000	0.99900	998.99	998.90	12.79	51.44	1.81	8.17	82.8
**Cocaine validation**	**Mean**	0.99655	0.96143	996.28	992.03	10.80	41.69	1.59	6.08	53.5
	**N**	175	175	175	175	175	175	175	175	175
	**Std. Deviation**	0.00774	0.03058	12.26	6.19	6.35	15.06	0.83	2.75	24.9
	**Minimum**	0.92130	0.78030	885.30	976.65	2.85	15.98	0.49	2.15	13.2
	**Maximum**	0.99980	0.99540	998.99	998.80	48.77	100.13	5.18	15.07	118.0
**Negatives; diasteriomers**	**Mean**	0.97735	0.94429	973.99	992.13	33.15	53.28	5.00	7.46	113.0
	**N**	10	10	10	10	10	10	10	10	10
	**Std. Deviation**	0.01061	0.02149	16.07	3.43	10.86	9.47	1.53	1.75	10.2
	**Minimum**	0.96050	0.90730	944.72	988.65	12.32	41.67	2.07	5.73	88.2
	**Maximum**	0.99710	0.96710	996.00	996.82	49.82	67.50	7.38	11.25	119.1
**Negatives; other**	**Mean**	0.40963	0.52040	379.22	298.63	86.87	141.36	10.39	21.15	124.0
	**N**	706	706	706	706	706	706	706	706	706
	**Std. Deviation**	0.24855	0.12737	315.68	134.80	16.32	14.24	3.68	2.17	1.0
	**Minimum**	0.06930	0.21670	8.51	67.77	50.63	101.24	7.28	14.93	122.3
	**Maximum**	0.94710	0.78210	913.93	499.10	148.50	158.18	24.10	24.37	126.4
**Total**	**Mean**	0.59010	0.65963	568.80	512.10	62.96	108.48	7.61	16.16	98.7
	**N**	1019	1019	1019	1019	1019	1019	1019	1019	1019
	**Std. Deviation**	0.34107	0.23505	387.55	339.83	38.64	51.59	5.22	7.85	41.4
	**Minimum**	0.06930	0.21670	8.51	67.77	1.08	7.62	0.18	1.07	3.9
	**Maximum**	1.00000	0.99900	998.99	998.90	148.50	158.18	24.10	24.37	126.4

*Using Stein's original weightings of x=0.6 and y=3 and equations 1 and 2.[1]

Table S3. Summary of binary classification figures of merit for various measures of spectral similarity for EASI and the consensus model for cocaine identification in the absence of retention time information relative to the cocaine training set. Only the 20 most abundant peaks in a spectrum are compared in each model, and the threshold is set to allow one false positive so that a likelihood ratio (TPR/FPR) can be calculated. The likelihood ratio is vastly underestimated because of the limited number of known negatives in the database. Mahalanobis distances are calculated for every spectrum relative the cocaine training set from Lab 1.

Model	Dot product		NIST Score		Euclidian distance		MAR		Mahalanobis distance
	EASI	Consensus	EASI	Consensus	EASI	Consensus	EASI	Consensus	
**Threshold**	0.982	0.967	991.1	996.2	28.00	43.62	4.48	5.74	102.20
**TPs**	297	220	297	142	298	232	299	220	298
**TNs**	715	715	715	715	715	715	715	715	715
**FPs**	1	1	1	1	1	1	1	1	1
**FNs**	6	83	6	161	5	71	4	83	5
**Sum**	1019	1019	1019	1019	1019	1019	1019	1019	1019
**FPR=**	0.1	0.1	0.1	0.1	0.1	0.1	0.1	0.1	0.1
**TPR=**	**98.0%**	**72.6%**	**98.0%**	**46.9%**	**98.3%**	**76.6%**	**98.7%**	**72.6%**	**98.3%**
**TNR=**	**99.9%**	**99.9%**	**99.9%**	**99.9%**	**99.9%**	**99.9%**	**99.9%**	**99.9%**	**99.9%**
**Accuracy**	**99.3%**	**91.8%**	**99.3%**	**84.1%**	**99.4%**	**92.9%**	**99.5%**	**91.8%**	**99.4%**
**+LR**	**702**	**520**	**702**	**335**	**704**	**548**	**706**	**520**	**704**

(1) Stein, S.; Scott, D. R., Optimization and testing of mass spectral library search algorithms for compound identification, *J Am Soc Mass Spectrom* **1994**, *5*, 859-866.
